# Quantifying the excess risk of infant mortality based on race/ethnicity at the county level to inform Michigan’s home visiting outreach plans

**DOI:** 10.1371/journal.pone.0203688

**Published:** 2018-09-12

**Authors:** Patricia McKane, Sarah Lyon-Callo, Nancy Peeler, Paulette Dobynes Dunbar, Brenda Fink

**Affiliations:** 1 Lifecourse Epidemiology and Genomics Division, Michigan Department of Health and Human Services, Lansing, Michigan, United States of America; 2 Bureau of Epidemiology and Population Health, Michigan Department of Health and Human Services, Lansing, Michigan, United States of America; 3 Infant and Early Child Health Section, Division of Family and Community Health, Michigan Department of Health and Human Services, Lansing, Michigan, United States of America; 4 Women, Infant, and Family Health Section, Division of Family and Community Health, Michigan Department of Health and Human Services, Lansing, Michigan, United States of America; 5 Division of Family and Community Health, Michigan Department of Health and Human Services, Lansing, Michigan, United States of America; Univesity of Iowa, UNITED STATES

## Abstract

**Objective:**

Michigan’s infant mortality rate is consistently higher than the national rate, with persistent and significant racial/ethnic disparities. In Michigan, nine counties account for more than 80% of all infant deaths. A home visiting program serving low-income, first-time mothers in high-risk communities is one strategy to reduce infant mortality. The objective of this study was to quantify the risk of infant mortality based on race/ethnicity within Michigan’s highest-risk counties to guide outreach for home visiting services in these counties.

**Methods:**

To maximize the efficiency of limited resources and to identify women at highest risk, we used decomposition to develop risk-based, county-specific estimates of excess infant deaths in nine Michigan counties using data from the 2007 to 2009 Michigan resident infant death file linked to the live birth/file.

**Results:**

The sample size for these counties was 200,610 live births and 1,836 infant deaths and for the reference population it was 195,180 live births and 1,133 infant deaths The study found that excess mortality varies among populations at the county level when compared to the reference population of infants born to Michigan mothers who attained more than a high school education and were at least 20 years of age at the infant’s birth. The excess risk of mortality was highest for African American infants in seven of the nine counties (56.5% to 132.8%) and for Hispanic infants (86.6%) and white infants (48.2%) in one county each.

**Conclusion:**

Even with a longstanding commitment and legal mandate to reduce disparities and with efforts to improve outreach into high-risk areas, disparities persist. An improved understanding of the racial/ethnic disparities within communities was useful to focus outreach efforts on reaching women at highest risk as part of subsequent program enrollment.

## Introduction

Infant mortality, defined as the death of an infant before the first birthday, is a globally accepted critical health indicator. The infant mortality rate (IMR), the number of infant deaths per 1,000 live births, may be a proxy for overall infant and maternal health, reflecting the health status of a community and access to and quality of pre- and postnatal maternal and infant medical care [1]. As such, infant mortality is not only an important indicator of maternal and child health but also a marker for the difficult-to-measure family- and community-level factors that affect the health of mothers and infants.

Although infant mortality in the United States has declined dramatically over the past century, the gap between African American and white infants persists and is projected to continue, even after controlling for marital status, educational attainment, and prenatal care [2,3]. Research has focused on examining the differences in infant mortality among African Americans and whites while accounting for low birthweight, premature birth, and maternal risk factors. Recently, Elder et al. concluded that a portion of the disparity persists and is unexplained by these characteristics [4]. Dominguez [5] has posited broadening the framework for studying racial/ethnic disparities in infant mortality from individual-level factors to societal factors, particularly racism, psychosocial stress, and socioeconomic factors.

**Fig 1:**
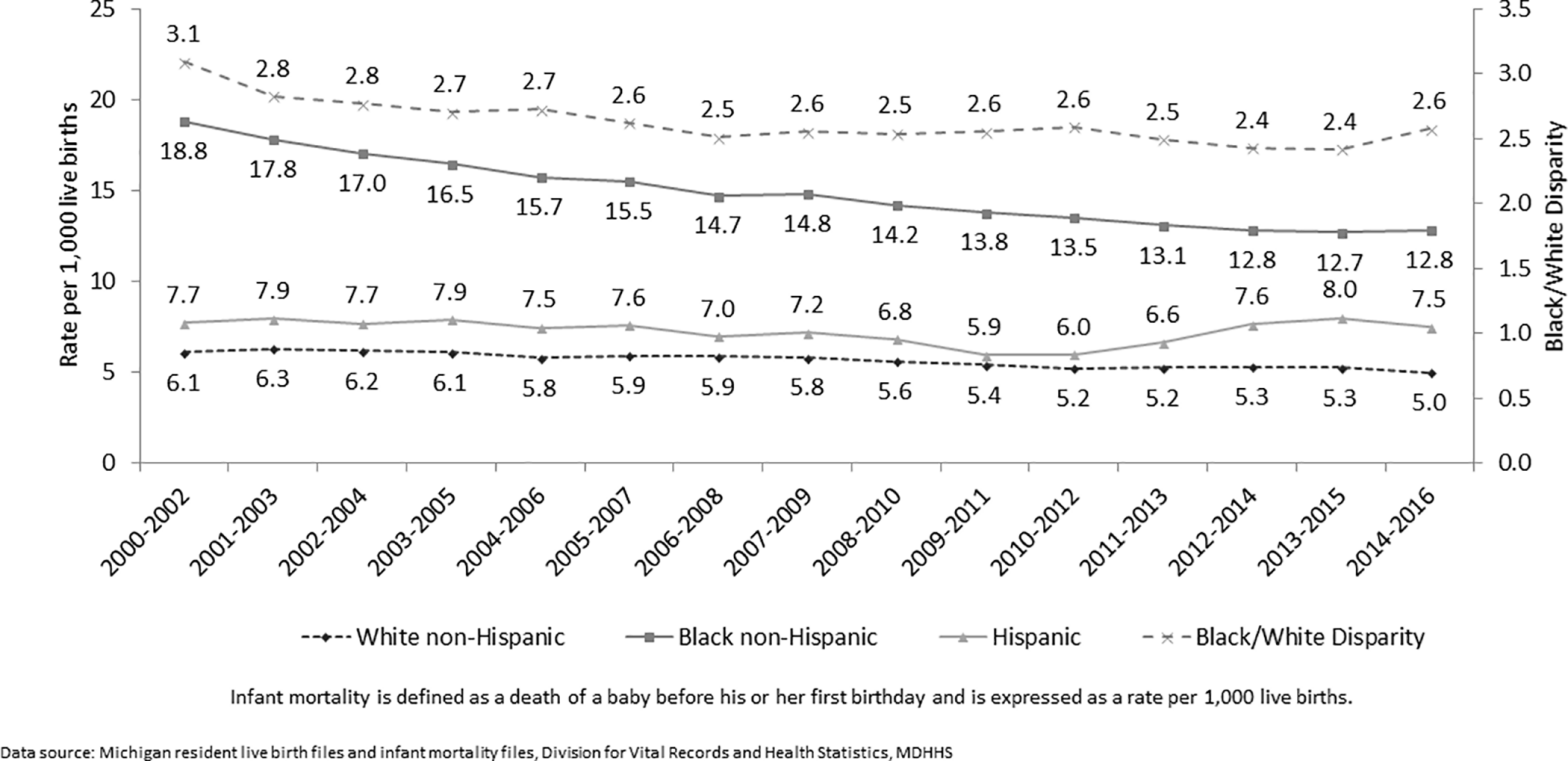
Trends in three year average infant mortality by race/ethnicity and disparities, Michigan 2000–2016.

Michigan’s IMR remains higher than the national rate (2016: 6.4/1,000 live births versus 5.9/1,000, respectively), and African American infants are more than twice as likely to die before their first birthday than white infants are [6]. Furthermore, infant mortality and racial disparities are not distributed equally throughout the state. Nine Michigan counties account for 80% of the state’s infant deaths and 84% of non-Hispanic African American (NH AA) infant deaths. Even with a longstanding commitment and a legal mandate to reduce disparities, they persist (Fig 1).

If Michigan is to reduce overall infant mortality, the state must address the disproportionate burden on high-risk communities and populations. Statewide IMR could be reduced by an estimated 30% if race/ethnicity-based disparities were eliminated. Home visiting is one strategy to improve birth outcomes, address the social determinants of health, and thereby reduce infant mortality. This effort was strengthened by a commitment to the Maternal, Infant, and Early Childhood Home Visiting (MIECHV) program, with its emphasis on infrastructure improvement and the use of evidence-based models, to improve critical outcomes for Michigan’s highest-need families with young children. The maternal and child health leadership at the Michigan Department of Health and Human Services (MDHHS) identified the MIECHV grant as an opportunity to expand an evidence-based home visiting program to aid in reducing persistent IMR disparities by addressing the underlying causes of disease across the whole population. These nine counties were selected for the expansion of home visiting programs to address the social determinants of health and improve birth outcomes.

Interventions and funds that are applied equally throughout the population, rather than targeted to high-risk individuals who lag behind the majority population, may work to widen racial/ethnic disparities in infant mortality [7]. Michigan MIECHV staff were concerned that despite intense efforts, racial disparities were persistent and home visiting program caseload demographics were not reflective of high-risk populations. As such, a targeted or directed population approach was planned for expansion of the home visiting program and involved focusing outreach on higher-risk groups or subpopulations.

One method used to examine infant mortality is the decomposition technique developed by Evelyn Kitagawa [8]. It has been used to decompose the effect of birthweight on infant mortality, to identify racial/ethnic differences in the teen birth rate by area-based poverty measures, and more recently to examine the excess risk of infant mortality in the Southern region of the United States [9, 10, 11]. The purpose of Kitagawa’s method is to explain the difference between two population rates and differences in their distribution [8]. The excess rate for a group compared to a reference population is parsed out into the portion attributable to the distribution differences versus the portion attributable to the specific rate differences. Decomposition, like standardization, is a well-established technique for comparing rates between groups whose demographic composition differs; however, decomposition allocates the rate difference into components. Decomposition addresses the additive contributions of differences in rates and distribution by strata in two populations to show the differences in their overall rates.

The purpose of this study was to quantify the excess risk of infant mortality within Michigan’s highest-risk counties compared to a reference population to guide outreach for home visiting program services.

## Methods

This study analyzes county-level infant death data linked to live birth data from Michigan between 2007 and 2009, the latest available data at the time of the study. County- or service area-level data provide a more accurate view of differences between racial/ethnic groups across counties, but the number of events in some of these counties is quite small and adding additional factors to the analysis would likely lead to cells with null values. While methods like Oaxaca-Blinder [12,13] with a non-linear extension [14] provide the advantage of the ability to model multiple predictors that could be contributors to the gap, the additional predictors were not needed for this analysis. In addition, regression based techniques would result in cells with null values or too few numbers to generate results for most of these nine counties. The Kitagawa method was used to analyze the differences between race/ethnicity-specific rates, while taking into account the differences in the distribution of race/ethnicity, at the county level to provide county estimates that are more useful for program planning.

### Data source

Data from the 2007 to 2009 Michigan resident live birth and infant death files linked to the birth files were used for this study. The analysis was restricted to the nine counties that account for more than 84% of Michigan’s NH AA infant mortality. This analysis was conducted using a dataset with limited identifiers (county, maternal race/ethnicity, year of death and maternal age) for the purposes of public health surveillance and program improvement. IRB approval was not sought for this study. A data use agreement was not required, but the author signed a confidentiality agreement and complied with all MDHHS data security and confidentiality policies.

### Analysis

The Kitagawa formula was used to calculate the excess risk of IMR by race/ethnicity for each high-risk county. Excess mortality is determined for each group within each county, based on comparing the race/ethnicity-specific IMR in the county to the race/ethnicity-specific IMR for the reference population and taking into account the county’s racial/ethnic distribution.

The reference population is Michigan women 20 years of age and older with more than 13 years of education and a live birth from 2007 to 2009. This group has lower IMRs, yet it is representative of Michigan women and thus represents attainable IMRs for high-risk communities. Income and poverty level are not available in the birth files; maternal educational attainment was used as a proxy.

**Fig 2:**
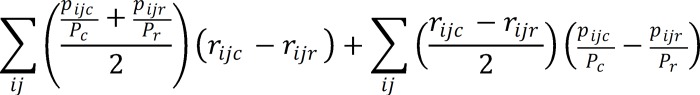
Kitagawa Formula. Where p_ijc/P_c = Rate in population ‘c’ p_ijr/P_r = Rate in the reference population r_ijc = Distribution in population ‘c’ r_ijr = Distribution in the reference population.

Excess mortality stratified by race/ethnicity is determined for each county by using the Kitagawa formula (Fig 2).

This formula was used to calculate stratum-specific excess mortality (SM) rates by comparing the IMR in the county to the rate in the reference population within each stratum and then summing the SM. For example, the average of the county and reference IMR is multiplied by the difference between the race/ethnicity distribution among live births in the county and reference populations for each race/ethnicity. This yields excess SM due to the difference in race/ethnicity distribution, under the assumption of average SM rates. The average of the county and reference race/ethnicity distribution among live births is multiplied by the difference between the county and the reference IMR. The result is excess SM due to the difference in SM, under the assumption of average distribution in that strata. The component due to the difference in distribution and the difference in SM are summed to yield the total excess combined excess mortality for each race/ethnicity strata.

The rate and percent excess mortality by race/ethnicity were calculated for each of the selected counties. The populations that contributed to the county’s excess mortality, compared to the reference population, were identified.

SAS 9.2 (SAS Institute Inc., Cary, NC, USA) was used for data management and analysis of live birth and infant mortality counts and Microsoft Excel was used to calculate the Kitagawa formula.

## Results

For the study period, racial/ethnic disparities in infant mortality are evident for each of the nine counties, the reference group, and the high-risk counties combined (Table 1). Infant mortality is highest among NH AA populations in each of the areas analyzed for this study. The analysis was completed for all racial/ethnic groups in each county but is not shown here. Among the NH AA population, the county-specific IMR ranges from 14.6 per 1,000 to 27.3 per 1,000; for the NH AA reference population, it is 14.4 per 1,000. Among NH whites, the IMR ranges from 4.5 per 1,000 to 9.1 per 1,000, with the reference population reporting a rate of 4.7 per 1,000. IMRs for the Hispanic population generally fall between the other two groups, the county-specific rates range from 5.9 per 1,000 to 12.2 per 1,000. The rate for the Hispanic reference population was 5.1 per 1,000.

**Table 1:** Infant mortality rate per 1,000 live births by race/ethnicity among Michigan high-risk counties, 2007–2009.

	NH White			NH AA			Hispanic		
	Rate	95% Confidence Interval		Rate	95% Confidence Interval		Rate	95% Confidence Interval	
County A	4.48	2.36	6.61	17.80	11.14	24.45	DNS		
County B	9.14	6.21	12.07	27.27	15.77	38.78	DNS		
County C	5.57	3.79	6.41	18.84	11.24	18.05	DNS		
County D	5.10	3.41	6.79	14.65	8.95	20.34	6.51	1.32	11.70
County E	5.18	3.47	6.89	23.57	16.06	31.07	8.65	1.10	16.20
County F	5.62	3.69	5.66	14.67	11.90	20.18	7.93	4.15	8.99
County G	4.67	3.88	5.47	16.04	12.86	19.22	6.57	3.36	9.78
County H	6.89	4.39	9.39	19.28	13.29	25.27	7.98	1.62	14.34
County I	6.21	5.36	7.07	17.06	15.70	18.43	7.03	4.91	9.15
Total High-Risk Counties	5.62	5.19	6.05	17.30	16.22	18.38	7.35	6.04	8.66
Reference	4.67	4.33	5.01	14.38	12.82	15.93	5.08	3.38	6.79

DNS: Data not sufficient for display. Cells with fewer than 5 deaths are suppressed.

The highest county-specific racial/ethnic disparity was a NH AA/NH white ratio of 4.6 in County E and the lowest (2.7) was found in County I. A disparity ratio of 2.7 was found in the reference group (calculated from Table 1).

Like the IMR, excess infant mortality was highest among NH AA in most counties but not all (Table 2). In some counties, excess mortality among NH white is expressed as a negative value, meaning that infant mortality among these women was lower than the state reference. The largest racial/ethnic disparity was seen in County G, where total excess mortality was highest among NH AA infants (132.8%) and lowest among NH white infants (-84.4%).

**Table 2:** Excess Mortality by components among high-risk Michigan counties, 2007–2009.

	**Actual (per 1,000) due to excess IMR**			**Percent (%) due to excess IMR **		
	NH White	NH AA	Hispanic	NH White	NH AA	Hispanic
County A	-0.1	0.6	DNS	-4.1	19.6	DNS
County B	3.4	1.7	DNS	51.4	2.8	DNS
County C	0.7	0.9	DNS	18.1	24.5	DNS
County D	0.3	0.0	0.1	22.6	2.8	6.4
County E	0.4	1.3	0.2	13.2	44.8	5.9
County F	0.7	0.0	0.3	56.2	3.0	22.0
County G	0.0	0.2	0.1	0.5	43.3	13.8
County H	1.5	1.0	0.2	30.9	19.7	4.1
County I	0.9	0.8	0.1	16.9	13.8	2.0
High Risk	0.6	0.6	0.1	19.4	17.4	3.9
	**Actual (per 1,000) due to distribution**			**Percent (%) due to distribution **		
	NH White	NH AA	Hispanic	NH White	NH AA	Hispanic
County A	-0.7	2.2	DNS	-21.7	68.3	DNS
County B	-0.2	0.6	DNS	-3.2	8.4	DNS
County C	-0.6	2.7	DNS	-17.1	75.5	DNS
County D	-0.5	0.7	0.3	-38.8	53.8	23.4
County E	-0.3	0.9	0.2	-11.8	32.7	6.4
County F	-0.6	0.2	0.8	-48.2	15.7	64.5
County G	-0.4	0.4	0.1	-84.9	89.5	28.9
County H	-1.2	2.7	0.4	-24.6	55.2	9.1
County I	-2.0	5.3	0.3	-35.8	95.6	4.8
High Risk	-1.1	2.6	0.3	-31.5	78.4	8.8
	**Total excess mortality (per 1,000)**			**Total excess mortality (%)**		
	NH White	NH AA	Hispanic	NH White	NH AA	Hispanic
County A	-0.8	2.8	DNS	-25.8	87.9	DNS
County B	3.2	2.2	DNS	48.2	33.5	DNS
County C	0.0	3.6	DNS	1.0	100	DNS
County D	-0.2	0.8	0.4	-16.2	56.5	29.8
County E	0.0	2.2	0.4	1.4	77.5	12.3
County F	0.1	0.2	1.1	8.0	18.7	86.5
County G	-0.4	0.7	0.2	-84.4	132.8	42.7
County H	0.3	3.7	0.6	6.3	74.9	13.2
County I	-1.0	6.0	0.4	-18.9	109.4	6.8
High Risk	-0.4	3.2	0.4	-12.1	95.8	12.7

DNS: Data not sufficient for display. Results based on less than 5 observations are suppressed

In county F, deaths of Hispanic infants accounted for the majority of excess infant mortality. This was not evident by comparing the unadjusted IMR by race/ethnicityfor this county but was discovered when the contribution of the distribution of race/ethnicity was calculated using the Kitagawa formula. In addition, the percent contribution to the excess mortality of smaller racial/ethnic groups was lower compared to unadjusted IMR (data not shown). Some of these results should be interpreted cautiously, as they are based on small numbers, but they also reflect the experience in these counties and identify high-risk groups that aggregate analysis ignores.

## Discussion

Despite improvement in IMR, racial/ethnic disparities persist (Fig 1). This paradox is the impetus behind the broadening of home visitation programs to reach vulnerable populations with an intervention aimed not only at improving birth outcomes but also at addressing some of the social determinants of health and thus having a lasting impact on the families enrolled. Like other public health priorities, strategies to reduce infant mortality can involve a population approach, a high-risk approach, a vulnerable-population approach, a directed-population approach, or a combination of these [15, 16, 17, 18]. In the population approach, public health measures are implemented to reduce the level of risk in the whole population and assumes that all groups are at equal risk and respond in the same way to interventions [17]. The high-risk approach focuses attention on sub-groups with high-risk exposure to specific risk factor [15] but doesn’t focus on upstream determinants that influence health behaviors. In contrast, a vulnerable population is a subgroup who has shared characteristics, such as socioeconomic status, that exposes them to higher risks [16]. The targeted or directed population approach involves focusing on higher risk groups or sub-populations [16–18].

Screening methods are not typically used to identify the higher-risk groups. Instead, epidemiological and/or sociodemographic data are used to define a particular subpopulation. For example, the high-risk approach has been predominating in the prevention of oral diseases, but now a combination of the high-risk and directed-population approaches is thought to be the better option [19]. The Marmot review of 2010 introduced the concept of “proportionate universalism,” which proposes that to reduce the steepness of the social gradient in health, interventions must be universal but with a scale and intensity proportionate to the level of disadvantage [20]. To integrate this concept into an intervention outreach strategy, it is necessary to identify subpopulations that are vulnerable or at high risk.

To achieve the goal of reducing infant mortality overall and especially among high-risk groups while maximizing limited resources, a directed-population approach was utilized in Michigan MIECHV planning. Standardization and decomposition are commonly used techniques to adjust for the confounding due to differences in the distribution of confounding factors or the population composition and are well suited to the purpose of guiding outreach efforts [21, 8, 22]. By its nature, decomposition does not imply a causal relationship but answers the question of how rates differ between the target population and reference population. Basically, it aims to answer the question: “What would the infant mortality rate be if the populations were the same?” Although there are many examples in the literature of the use of decomposition analysis, few were used to inform program outreach. In this study, decomposition of infant mortality at the county level by race/ethnicity identified high-risk groups who were overlooked when the analysis was limited to IMR with races/ethnicities of less populous groups aggregated into “other.” Furthermore, results for County F were unique, in that the highest excess risk was found among Hispanic infants, not NH AA as is found for the other high-risk counties. The local health department verified that infant mortality among Hispanics was an emerging issue in County F. Follow-up analysis (both quantitative and qualitative), including work with the Hispanic community to better understand the needs and identify solutions, is underway in this county.

This study is unique; the findings are being used to guide home visiting program outreach to high-risk populations within these individual counties. Demographic data of program participants are collected by the home visiting program and reported to the state. In some of these counties enrollment and outreach was not representataive of high risk groups. Based on the results of this analysis, each local agency developed, submitted and implemented an outreach plan to reach populations with the highest risk of excess infant mortality. Additional funding was made available to the sites to support specific outreach activities. These activities included direct contact, education and relationship building with providers, schools, churches and potential clients. Some agencies used media campaigns and engaged community partners. In County F, where excess moratality was highest amongst Hispanic infants, the local agency worked closely with health centers serving the Hispanic community and sought to hire home visitors fluent in Spanish.

Despite the importance of addressing racial/ethnic health disparities, the authors are not aware of other published literature demonstrating the development of an outreach plan based on decomposition analysis stratified by race/ethnicity. Basing enrollment on race/ethnicity alone, even when based strictly on risk, may raise discrimination concerns. Addressing health disparities can be achieved by identifying high-risk populations and ensuring that they are being included in program outreach efforts. Currently, MDHHS is using this analysis to guide outreach plans at the state and local levels.

There are several limitations to this study. First, despite combining three years’ worth of data, the use of county-level data resulted in small cells in the number of infant deaths for some racial/ethnic groups. Small numbers are suppressed for publication to protect confidentiality, yet for program and surveillance purposes, it is necessary to monitor the health outcomes among smaller populations, who are often at higher risk for an adverse outcome. While it is true that mortality or disease rates in small populations can change dramatically based on only one or two additional cases (hence the label “unstable”), events happening within these groups shouldn’t be ignored. Traditional methods of addressing small numbers include combining multiple years’ worth of data, collapsing categories, and broadening geographical boundaries. However, this masks a geographic or demographic subgroup which has high mortality. Also, this study is used to inform program outreach to high-risk populations within a defined geographic area (county or large city), so it is necessary to analyze data at this level. Furthermore, it is important to analyze deaths by all racial/ethnic groups and not simply choose the most populous, as many small groups bear a disproportionately high risk. By decomposing by race/ethnicity, we account for the contribution relative to the distribution of race/ethnicity, yielding an estimate of the excess mortality for all racial/ethnic groups within each county. Although categorization of race/ethnicity may be less than perfect, it is collected consistently throughout the counties during this time period.

Secondly, the data are from 2007–2009. However, these were the most recent data available at the time of the analysis, conducted in 2012.

In addition, the selection of the reference population is important for any standardization or decomposition technique. In this study, the reference population was selected to most closely resemble what infant mortality could look like in Michigan for the same period. Parity was considered as a factor in the reference population since the intervention is limited to first-time mothers, but this was rejected for several reasons. The Michigan Resident Live Birth file includes the number of prior live births and stillbirths, but these fields are self-reported and subject to bias. Currently, the numbers to analyze infant mortality by race/ethnicity at the county level among first-time mothers are not robust enough for this analysis, but this is something being considered for future analysis.

### Public health implications

In this study, data analysis developed uniquely for each county, reflected the excess risk rate of infant mortality, taking into account the difference in race/ethnicity within each county compared to the standard population. Results were shared with local health departments to encourage outreach to underserved communities.

Although the analytic technique is commonly used and not controversial, resistance was encountered when the results were shared. Previously, geographic-based enrollment guidelines for perinatal home visiting programs failed to increase enrollment among racial/ethnic minorities. Furthermore, limiting enrollment to densely populated urban areas ignores women residing in rural areas of these counties. During the recession, Michigan’s population declined, particularly among young women, and the population within the state shifted out of urban areas.

There is agreement that disparities are unacceptable and that the highest-risk populations should be prioritized to receive services. Yet, despite our efforts, disparities persist, and limiting services to high-risk areas (based on geography) has not yet resulted in the improved enrollment of high-risk groups. Using decomposition analysis is a different approach, and although its effectiveness has yet to be determined, it has led to increased collaboration between epidemiologists and program managers and between state and local health departments. Guidance to communities and agencies is a dynamic and collaborative process. Some agencies embraced the analysis from the start; others were resistant; but more recently, all have adopted it to guide their outreach. Over time, the response has been positive, as the analysis has led to outreach to groups who may have been overlooked in the past.
